# Assessing the Accuracy and Readability of Large Language Model Guidance for Patients on Breast Cancer Surgery Preparation and Recovery

**DOI:** 10.3390/jcm14155411

**Published:** 2025-08-01

**Authors:** Elena Palmarin, Stefania Lando, Alberto Marchet, Tania Saibene, Silvia Michieletto, Matteo Cagol, Francesco Milardi, Dario Gregori, Giulia Lorenzoni

**Affiliations:** 1 Unit of Biostatistics, Epidemiology and Public Health, Department of Cardiac, Thoracic, Vascular Sciences and Public Health, University of Padova, 35131 Padova, Italy; elena.palmarin@ubep.unipd.it (E.P.); stefania.lando@ubep.unipd.it (S.L.); giulia.lorenzoni@unipd.it (G.L.); 2 Breast Surgery Unit, Veneto Institute of Oncology IOV, IRCCS, 35128 Padova, Italy; alberto.marchet@iov.veneto.it (A.M.); tania.saibene@iov.veneto.it (T.S.); silvia.michieletto@iov.veneto.it (S.M.); matteo.cagol@iov.veneto.it (M.C.); 3 General Surgery, Department of Surgery, Oncology and Gastroenterology, University of Padova, 35128 Padova, Italy; milardi94@gmail.com

**Keywords:** large language models, patient education, breast cancer, perioperative care

## Abstract

**Background/Objectives:** Accurate and accessible perioperative health information empowers patients and enhances recovery outcomes. Artificial intelligence tools, such as ChatGPT, have garnered attention for their potential in health communication. This study evaluates the accuracy and readability of responses generated by ChatGPT to questions commonly asked about breast cancer. **Methods:** Fifteen simulated patient queries about breast cancer surgery preparation and recovery were prepared. Responses generated by ChatGPT (4o version) were evaluated for accuracy by a pool of breast surgeons using a 4-point Likert scale. Readability was assessed with the Flesch–Kincaid Grade Level (FKGL). Descriptive statistics were used to summarize the findings. **Results:** Of the 15 responses evaluated, 11 were rated as “accurate and comprehensive”, while 4 out of 15 were deemed “correct but incomplete”. No responses were classified as “partially incorrect” or “completely incorrect”. The median FKGL score was 11.2, indicating a high school reading level. While most responses were technically accurate, the complexity of language exceeded the recommended readability levels for patient-directed materials. **Conclusions:** The model shows potential as a complementary resource for patient education in breast cancer surgery, but should not replace direct interaction with healthcare providers. Future research should focus on enhancing language models’ ability to generate accessible and patient-friendly content.

## 1. Introduction

Breast cancer is among the most prevalent malignancies affecting the global female population and remains the leading cause of cancer-related mortality worldwide. Global breast cancer statistics indicate 2.3 million new cases of breast cancer in 2020, accounting for about 12% of incident cancer cases and resulting in 685.000 deaths [1]. At the European level, more than 374,000 new cases of breast cancer were reported in 2022, accounting for more than 29% of all new cancer diagnoses [2]. These figures underscore the centrality of surgical treatment in breast cancer care and the need for accessible, high-quality perioperative information for patients.

The impact of this disease extends beyond the physical realm, profoundly affecting the psychological well-being of patients who face complex challenges throughout the diagnostic and therapeutic journey. The perioperative period is a particularly vulnerable phase of the breast cancer care journey. Patients are often called upon to make quick and emotionally charged decisions while facing complex medical information. Recent literature has also emphasized the importance of robust assessment methods in the context of breast reconstruction, particularly with emerging techniques, such as fat grafting [3], which require clear and accessible information to support patient decision-making.

In this context, the ability to access timely, reliable, and clearly presented information becomes a cornerstone of shared decision-making and patient-centered care [4]. In addition, disparities in health literacy can exacerbate inequities in care outcomes, underscoring the importance of personalized and accessible communication tools. Evidence suggests that patients’ improved understanding of their condition, treatment plans, and recovery processes enhances satisfaction, alleviates excessive anxiety, and fosters greater engagement in the clinical pathway [5].

With the rise of digital technologies, artificial intelligence (AI) has become a promising resource for addressing patient information needs. Large language models (LLMs), particularly conversational AI systems built on LLMs like ChatGPT, have gained attention as potential tools for delivering health-related information [6,7,8]. Their applications in healthcare have been explored across various contexts, including clinical decision support, medical documentation, answering medical queries, and patient education [9,10].

Importantly, as LLMs are increasingly incorporated into publicly available tools, patients may turn to these systems for guidance before or after consulting a medical professional. This trend raises both opportunities and concerns. On the one hand, these tools could help fill information gaps, especially when access to health professionals is limited. On the other hand, the lack of human supervision, variability in content quality, and limited ability to provide personalized responses call for systematic evaluation of these tools, especially when used in sensitive settings, such as cancer surgery. The reliability of these systems as a source of medical information—particularly in the sensitive context of breast cancer—remains a subject of growing debate [6,11,12]. While some studies highlight limitations, such as inconsistencies and contradictory outputs, others raise concerns about the information’s accuracy, privacy, bias, and legality. Additionally, questions persist regarding the systems’ ability to fully understand user questions and deliver easily understandable answers [13,14].

Understanding how these models perform in realistic, patient-centered scenarios can help define their appropriate role within clinical workflows. Rather than acting as stand-alone sources of medical advice, language templates, such as ChatGPT, could be integrated as support tools that reinforce information provided during consultations, clarify perioperative instructions, and offer accessible summaries of key recommendations. For example, they could be used to generate patient handouts, discharge instructions, or customized digital FAQs for common issues in breast cancer surgery. These tools can be particularly helpful in settings where time constraints limit in-person explanations or when patients require additional reassurance upon returning home. In this way, LLMs can help bridge communication gaps and help make patients more informed, engaged, and confident, without replacing the essential human relationship at the heart of surgical care.

In light of these considerations, the present study aims to evaluate the accuracy and readability of the conversational version of OpenAI’s GPT (ChatGPT) in generating responses to questions commonly asked by breast cancer patients regarding perioperative care, particularly about preparation for and recovery from surgery.

## 2. Materials and Methods

**Figure 1:**
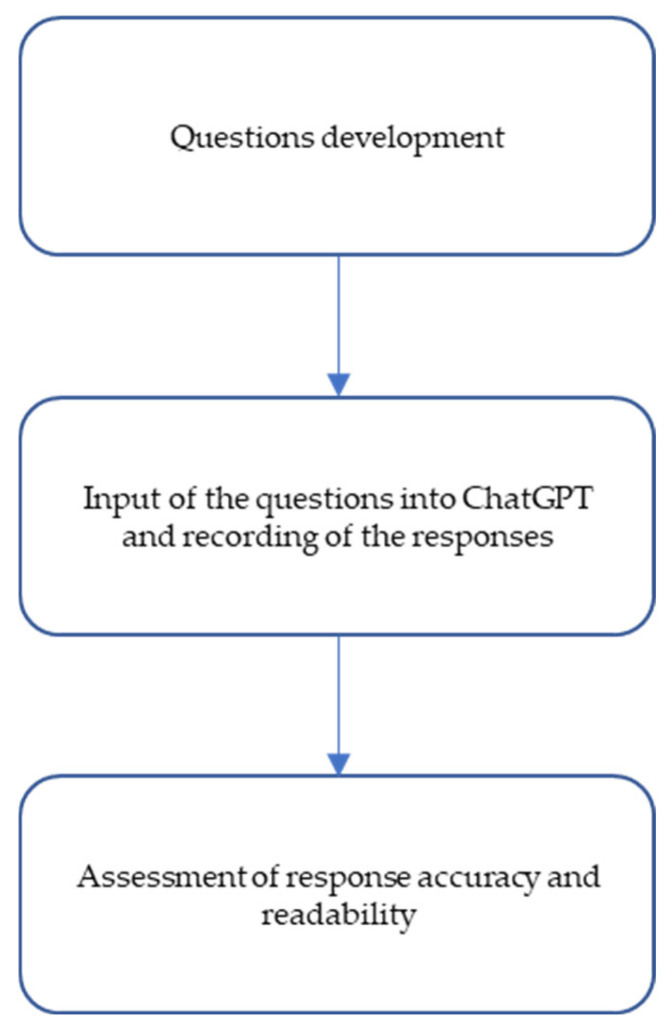
Methodological flowchart.

This observational study was conducted in November 2024. Fifteen questions, designed to represent queries from breast cancer patients, were submitted to ChatGPT (4o version). The questions concerned preparation for and recovery from surgery. Using REDCap’s “survey” feature, questions were administered anonymously to a group of 35 breast surgeons. Of these, 29 completed the survey, and their responses were included in the analysis. The readability was assessed using a readability tool accessed through a web-based application. Figure 1 presents the methodological workflow.

### 2.1. Questions

Drawing on clinical guidelines, clinical experience, scientific materials related to preoperative education, and printed educational brochures (based on national and international guidelines) routinely used, 15 questions were developed to simulate those of breast cancer patients, focusing on perioperative care and emphasizing preparation for and recovery from surgery. The topics addressed by these questions included smoking cessation, fasting, preoperative tests, medication management, skin preparation and shaving before surgery; wound care, drainage management, return to physical activity, lymphoedema prevention, nutrition, and pain management after surgery; as well as the psychological impact of the procedure, its effects on intimate and sexual life, the expected duration of hospitalization, and the need for follow-up care. The complete list of questions can be found in Supplementary Table S1.

To minimize biases and ensure consistency in ChatGPT’s responses, each question was submitted to the chat using a standardized prompt. The prompts explicitly specified that they came from women with breast cancer preparing for surgery, aiming to replicate realistic patient queries. Each question began with the standardized sentence “I need surgery for breast cancer” to provide context and ensure uniformity.

### 2.2. ChatGPT and Response Generation

ChatGPT was chosen for this study because it currently represents the most widely adopted and recognizable chat-based LLM, particularly in the biomedical field. Its public availability, ease of use, and integration with common communication platforms make it the most easily accessible tool for patients seeking health information. The 4o version was employed, which provides notable advancements in human–computer interaction, offering superior capabilities in processing multimodal inputs and outputs compared to previous models and improved performance in tasks involving vision and audio understanding.

Each question was entered only once as a separate and independent prompt using the ‘New Chat’ function. In this way, we ensured that no contextual memories or previous conversations influenced the model’s response, minimizing carry-over effects and ensuring consistency between interactions. The responses generated were documented alongside their corresponding questions in a Microsoft Word file for subsequent evaluation.

The detailed answers can be found in Supplementary Table S1.

### 2.3. Grading: Accuracy and Readability

A pool of breast surgeons independently evaluated the accuracy of each response. All the surgeons had expertise in breast surgery and in providing patients with information regarding preparation for and recovery from surgery. For each question, the final accuracy rating was determined according to the mode of the assigned scores.

Drawing on a thorough literature review, the evaluations were conducted using a standardized 4-point Likert scale, defined as follows: (1) comprehensive: accurate and complete, with no additional information a board-certified specialist would add if asked this question by a patient; (2) correct but incomplete: all information is correct but incomplete; a board-certified specialist would have more important information to add if asked this question by a patient; (3) some correct and some incorrect; (4) completely incorrect [11].

The readability of the responses was assessed using the Flesch–Kincaid Grade Level (FKGL), calculated using an online tool (https://readable.com, Added Bytes Ltd., Brighton, England). This tool was chosen because of its widespread use in similar studies, which allows for consistent comparison of results throughout the literature [11,15].

The Flesch–Kincaid score objectively measures text complexity by analyzing three key factors: sentence length, word difficulty, and textual structure. The resulting U.S. grade level score indicates the minimum educational attainment required to comprehend the text. Scores range from 0 to 18, with higher values representing more complex texts [16]. For instance, a score of 5.3 implies that fifth-grade students can easily understand the text. This standardized approach ensures a clear and consistent evaluation of textual accessibility, particularly relevant in healthcare settings.

In this study, the online tool was applied to each of the 15 responses generated by the LLM, providing the following:

- A numerical score reflecting the minimum level of schooling required to understand the text.
- A qualitative classification describing the degree of reading difficulty.
- The corresponding educational level (e.g., elementary school, university) within the U.S. school system [16].

In healthcare communication, patient education materials are recommended to target a Flesch–Kincaid Grade Level score of 6 to 8. This ensures the information is accessible to a wide audience, including individuals with varying educational backgrounds [17].

### 2.4. Statistical Analysis

Descriptive statistics were reported as the median (I–III quartiles) for continuous variables related to readability and as absolute numbers (percentages) for categorical variables related to accuracy. Analyses were performed using Jamovi software (version 2.3.28.0).

## 3. Results

ChatGPT’s responses were rated as “accurate and comprehensive” (score of 1 on the 4-point Likert scale) for 11 out of 15 questions (73%), while 4 out of 15 answers (27%) were rated as “correct but incomplete” (score of 2 on the scale). Notably, none of the responses was classified as “partially incorrect” or “completely incorrect”, corresponding to scores of 3 and 4. The four answers rated as correct but inadequate pertained to the drainage management, psychological wellbeing, duration of hospitalization, and the need for postoperative follow-up.

Readability analysis revealed high FKGL scores for the ChatGPT-generated responses, with a median of 11.2 (10.0,11.8). Table 1 presents the correspondence between the Flesch–Kincaid scores and the corresponding educational levels.

An FKGL score of 11.2 corresponds to a high school reading level and indicates that at least eleven years of education are required to understand the text. Notably, only one response related to postoperative wound care achieved a readability score of 8.7, placing it within the first quartile and indicating a basic reading difficulty. In contrast, the responses addressing the resumption of physical activity, the duration of hospitalization, and the need for postoperative follow-up had FKGL scores of 12.4, 13.6, and 13.3, respectively, exceeding the third quartile and corresponding to an advanced reading level. Table 2 provides a summary of the accuracy and readability of ChatGPT-generated responses. Furthermore, the detailed accuracy and readability scores for all responses are available in Supplementary Table S2.

## 4. Discussion

The results of the present study demonstrate that the LLM model provided “accurate and comprehensive” responses to most of the answers, a proportion consistent with findings from previous studies on similar applications in oncological and surgical contexts. For instance, Shao et al. evaluated the use of ChatGPT for perioperative education in thoracic surgery, reporting an appropriateness rate of 92% of answers, which highlights the potential of such tools to improve patient education [9]. Similarly, Moazzam et al. examined the quality of ChatGPT’s responses to questions about pancreatic cancer and its surgical management, finding that 80% of responses were rated as “very good” or “excellent” [18]. Regarding specifically breast cancer, several published studies have evaluated the accuracy of ChatGPT in answering questions on the topic, even if not explicitly related to perioperative care. Some studies have focused on prevention and screening [19,20]. In contrast, others have examined breast reconstruction [6,21], conditions associated with breast implants [22], or more general topics ranging from risk factors to treatment and prognosis [11,23,24,25,26,27]. Except for a few exceptions, such as the study by Park et al. [11], which was conducted using an earlier version of ChatGPT (version 3.5), generally, the results were encouraging, further supporting the potential role of LLMs in this clinical area. For example, ChatGPT has been shown to outperform Google as a source of information for patients, further confirming the potential of LLMs to improve the quality of healthcare communication [24]. These findings, combined with the high accuracy of the responses provided by the model in this study, confirm that ChatGPT can serve as a valuable informational resource and a helpful tool to complement health education across various clinical fields, including breast cancer surgical oncology.

However, the study highlighted some limitations of ChatGPT in providing adequate answers to questions about the length of hospital stay or postoperative follow-up, which were often rated as “correct but incomplete.” Discrepancies and inaccuracies, though rare, remain a critical concern, as previously noted in the literature. Prior studies have emphasized that ChatGPT may generate incorrect or incomplete information, limiting its reliability in clinical contexts without human oversight [13,14]. Therefore, viewing the model as an integrative tool rather than a substitute for healthcare professionals’ expertise and clinical judgment is essential, particularly for sensitive topics, such as preoperative and postoperative care in surgical procedures. Finally, although this study focused exclusively on the textual characteristics of ChatGPT responses, it is important to recognize that factors such as patients’ emotional states, cognitive load after surgery, and the use of medications or supplements may influence how information is processed and understood [28]. Future research should consider these issues when evaluating the real-world applicability and impact of AI-generated health communication.

Another key aspect emerging from this study concerns the readability of responses. The median readability level of responses provided by the model, measured using the Flesch–Kincaid Grade Level (FKGL), was 11.2, significantly higher than international recommendations, which suggests a target level between 6 and 8 for patient-directed materials [11,21]. Although each question was explicitly framed from a patient’s perspective, with every prompt beginning with the phrase “I need surgery for breast cancer”, the responses often used technical terms and complex language, making the information less accessible to a lay audience. Clear and simple language ensures that patients fully understand the information and make informed decisions. Furthermore, studies have shown that overly complex informational materials not only reduce treatment adherence but can also increase anxiety levels in patients, compromising the effectiveness of health education and information delivery [20]. While ChatGPT is technically capable of simplifying language upon request, this requires active interaction, which is not always straightforward or feasible for all patients [20].

### 4.1. Future Directions

Future studies should focus on how tools like ChatGPT can be effectively utilized in real-world clinical settings to support patient education. It will be important to investigate whether these tools help patients better understand instructions before and after surgery, follow medical advice more closely, and feel more supported during care. Research should also investigate how to improve the way questions are asked to the model so that the answers are easier to understand and more relevant to each situation. Another useful direction would be to develop sets of ready-to-use questions and answers prepared by health care providers that can be safely shared with patients. Finally, ChatGPT and similar tools could be integrated into existing systems, such as hospital websites, patient apps, or printed materials, to make high-quality information more accessible, especially in settings where physicians and nurses have limited time.

### 4.2. Implications for Clinical Practice

Integrating tools such as ChatGPT into clinical practice could offer practical benefits at specific stages of the care pathway, such as the period between the surgical visit and hospital admission, or during preparation for discharge. For example, patients often forget some of the information discussed during in-person visits; providing access to a reliable digital tool could help reinforce key instructions at home. In addition, in busy outpatient settings or during high-volume surgical activity, these tools could help staff handle frequently asked questions, reducing repetitive explanations and freeing up time for more personalized discussions.

If carefully integrated into existing workflows, these technologies can contribute to more efficient communication, better patient preparation, and more sustainable use of clinical resources. However, it is essential to emphasize that while LLMs show promise in supporting patient education, their role should enhance, not replace, interactions with healthcare professionals. Integrating such tools can help alleviate the educational burden on healthcare staff, particularly in resource-limited settings. Nonetheless, supervision by healthcare professionals is essential to ensure the relevance and accessibility of the information provided. This can be achieved by integrating LLM-generated content into existing patient education workflows, where clinicians can review and, if necessary, adapt information before it is delivered to patients, ensuring consistency with clinical guidelines and individual patient needs. However, as patients increasingly access ChatGPT independently, such oversight cannot always occur at the source. In these cases, it is crucial that healthcare providers actively engage with the information patients bring to the consultation, verifying its accuracy, addressing any misunderstandings, and situating it within the appropriate clinical context. This form of ex-post supervision is a pragmatic approach to mitigate the risks of unsupervised AI-generated content in actual clinical practice.

## 5. Conclusions

This study demonstrates the potential of LLM as a complementary tool for providing accurate and comprehensive information to patients undergoing breast cancer surgery. These findings are consistent with previous research in oncological and surgical contexts, confirming its value as an informational resource in clinical practice.

In conclusion, this study represents an additional step in exploring the capabilities of LLM as an informational tool in clinical patient education, particularly for preoperative and postoperative education in women undergoing surgical treatment for breast cancer. Its potential as a valuable complement to traditional healthcare communication is evident, but its application requires a thoughtful and patient-centered approach to ensure safety and reliability.

## Figures and Tables

**Table 1:** Relationship between the Flesch–Kincaid Grade Level score, readability level, and educational level (Flesch Reading Ease and the Flesch–Kincaid Grade Level, s.d.).

Flesch-Kincaid Score	Reading Level	School Level
0–3	Basic	Kindergarten/Elementary
3–6	Basic	Elementary
6–9	Average	Middle School
9–12	Average	High School
12–15	Advanced	College
15–18	Advanced	Post-grad

**Table 2:** Answers’ accuracy and readability. Data are median (I, III quartiles) for continuous variables and absolute numbers (percentages) for categorical variables.

	N	ChatGPT-4o (N = 15)
FKGL	15	11.2 (10.0, 11.8)
Accuracy	15	
Comprehensive		11 (73%)
Correct but incomplete		4 (27%)

## Data Availability

The original contributions presented in this study are included in the Supplementary Material.

## Supplementary Materials

The following supporting information can be downloaded at https://www.mdpi.com/article/10.3390/jcm14155411/s1: Table S1. List of fifteen questions designed to represent breast cancer patients’ concerns about surgical preparation and recovery, and answers generated by ChatGPT-4o. Table S2. Accuracy (4-point Likert scale) and readability (FKGL) scores for each individual response generated by ChatGPT-4o.
