# Supplementary material for: Assessing the Accuracy and Readability of Large Language Model Guidance for Patients on Breast Cancer Surgery Preparation and Recovery
Source: J Clin Med. 2025 Aug 1;14(15):5411. doi: 10.3390/jcm14155411 (PMC12347998; doi:10.3390/jcm14155411)
Supplement: Supplementary file 1 [file jcm-14-05411-s001.zip › jcm-3707750-supplementary.pdf]

Supplementary Material

Table S1. List of fifteen questions designed to represent breast cancer patients' concerns about surgical preparation and recovery and answers generated by ChatGPT-4o.

Question	ChatGPT-generated responses
**1. I need surgery for breast cancer. Can I continue smoking before and after the procedure?**	I'm sorry to hear about your diagnosis. Smoking is generally discouraged before and after any surgery, including breast cancer surgery, as it can increase risks and slow down healing. Here are some key points to consider:<br><br>1. Preoperative Period: Smoking reduces blood flow and oxygen supply to tissues, which can interfere with the body's ability to heal. Quitting smoking in the weeks leading up to surgery can reduce these risks. Ideally, it's recommended to stop smoking at least 4-6 weeks before the procedure, as this can significantly improve surgical outcomes.<br>2. Postoperative Recovery: After surgery, smoking can increase the risk of complications, such as infections, poor wound healing, and even delayed recovery. Smoke exposure can also suppress the immune system, making it harder for your body to recover fully.<br>3. Long-term Health: Smoking is also linked to a higher risk of cancer recurrence and other health issues. Quitting can improve your overall prognosis and support your body in healing and fighting any residual cancer cells.<br><br>Consider reaching out to your healthcare team about resources to help with smoking cessation. They can guide you to programs, medications, or counseling to support you in this process.
**2. I need surgery for breast cancer. What am I allowed to eat and drink beforehand? Do I need to fast?**	Before surgery, you will need to follow specific instructions regarding eating and drinking to minimize risks and prepare your body safely. Here's what is generally recommended:<br><br>1. Fasting Guidelines:<br>• Solid Foods: You will likely need to stop eating solid foods at least 8 hours before your scheduled surgery. This includes all foods and snacks, as they take longer to digest.<br>• Clear Liquids: You might be allowed to drink clear liquids up to 2 hours before surgery. Clear liquids include water, clear juices (like apple juice), and clear broth. Avoid anything with milk

	or pulp.<br>2. Avoiding Certain Foods and Beverages:<br>• Dairy Products: Avoid milk, cream, and other dairy products on the day of your surgery, as they can slow digestion.<br>• Heavy or Fatty Foods: Refrain from high-fat foods for 24 hours before surgery since they take longer to digest and can interfere with anesthesia.<br>• Alcohol and Caffeine: Avoid alcohol and limit caffeine the day before, as these can affect hydration and anesthesia effects.<br>3. Medication and Supplements:<br>• Certain medications or supplements, especially blood thinners or herbal supplements, might need to be adjusted or stopped before surgery. Always check with your healthcare team regarding any medications you're taking.<br><br>It's essential to follow the specific instructions given by your surgical team, as individual requirements can vary based on the type of anesthesia and your health conditions.
**3. I need surgery for breast cancer. What pre-operative tests will I need to complete?**	For breast cancer surgery, your healthcare team will likely recommend several pre-operative tests to ensure your body is prepared for the procedure and to minimize potential risks. Common pre-operative tests include:<br><br>1. Blood Tests:<br>• Complete Blood Count (CBC): Assesses your overall health and checks for conditions like anemia or infection.<br>• Electrolytes and Kidney Function: Measures levels of key electrolytes and evaluates kidney health.<br>• Blood Clotting Tests: Includes PT, PTT, or INR tests to check how well your blood clots, as some medications or conditions may affect bleeding risk.<br>2. Imaging Tests:<br>• Chest X-ray: Evaluates lung health and screens for any issues that may affect anesthesia.<br>• Electrocardiogram (ECG): Monitors heart rhythm and function, especially if you have a history of heart disease or if you are over a certain age.<br>3. Diagnostic Imaging:<br>• Mammogram or Breast MRI: Additional imaging may be needed to verify the location and size of

	the tumor before surgery, especially for certain breast cancer types.<br>4. Additional Tests Based on Health History:<br>• If you have any other medical conditions (e.g., diabetes, hypertension), your doctor may order tests to ensure those are well-managed before surgery.<br><br>Your surgical team will review all results to tailor the anesthesia and surgical approach to your needs, making sure everything is optimized for your safety and recovery. Always consult your doctor for personalized advice based on your unique health needs and surgical plan.
**4. I need surgery for breast cancer. Can I take medication before the operation?**	Before undergoing breast cancer surgery, it's essential to discuss all medications, supplements, and over-the-counter drugs you're taking with your healthcare team. Certain medications may need to be paused or adjusted prior to surgery to minimize potential risks. For instance, blood-thinning medications, such as aspirin or anticoagulants, are often stopped up to seven days before surgery to reduce bleeding risks.<br><br>Additionally, supplements like fish oil, vitamin E, and certain herbs (e.g., garlic, ginseng, gingko biloba, and St. John's Wort) may need to be discontinued, as they can increase bleeding tendencies.<br><br>It's crucial to provide your surgical team with a comprehensive list of all substances you're consuming, including:<br><br>• Prescription medications<br>• Over-the-counter drugs<br>• Vitamins<br>• Herbal supplements<br><br>This information allows your healthcare providers to offer personalized guidance on which medications to continue, adjust, or temporarily stop before your surgery. Always consult your doctor before making any changes to your medication regimen to ensure a safe surgical experience.
**5. I need surgery for breast cancer. How should I prepare my skin and shave before the procedure?**	Preparing your skin properly before breast cancer surgery is essential to reduce the risk of infection and ensure the best surgical outcomes. Here's a detailed guide based on current clinical recommendations:<br><br>Skin Preparation<br><br>1. Showering:<br>• Use an antimicrobial soap (chlorhexidine is often

	recommended) to shower the night before and the morning of surgery. Pay special attention to the area around your chest, underarms, and any other area specified by your surgical team.<br>• Avoid using lotions, creams, or powders on your skin after washing, as they can interfere with the sterile environment during surgery.<br>2. Avoid Skin Irritants:<br>• Do not use deodorants, perfumes, or antiperspirants in the days leading up to surgery, especially on the day itself, as they can leave residues that may interfere with the procedure.<br>3. Avoid Sun Exposure:<br>• Protect your skin from sunburn or irritation in the weeks leading up to surgery. Damaged skin may increase the risk of complications during recovery.<br><br>Shaving Guidelines<br><br>1. Do Not Shave Near the Surgical Area:<br>• Avoid shaving your underarms or the area where the incision will be made for at least 48 hours before surgery. Shaving can create micro-cuts that increase the risk of infection.<br>2. Follow Surgeon's Instructions:<br>• Some surgeons may ask you to avoid shaving entirely before surgery to minimize risks. Always clarify with your surgical team if you're unsure.<br><br>Additional Recommendations<br><br>1. Report Skin Issues:<br>• Inform your surgeon if you have any skin irritation, rash, or infections near the surgical site before the procedure.<br>2. Wear Clean, Comfortable Clothing:<br>• On the day of surgery, wear loose-fitting, clean clothing that is easy to remove and avoids irritation to the chest area.<br><br>By following these steps, you can help ensure a smooth surgical experience and recovery process. Always consult with your healthcare team for specific instructions tailored to your case.
**6. I need surgery for breast cancer. How should I care for my wound during the postoperative period?**	Caring for your wound properly after breast cancer surgery is essential for promoting healing and preventing infection. Here are some general tips:

1. Keep the Wound Clean and Dry

- Follow your healthcare provider's instructions on when it is safe to shower, typically a few days post-surgery.
- Use a gentle, non-scented soap when cleaning around the wound area, and pat dry with a clean towel. Avoid scrubbing the incision directly.
- Until your doctor says it's okay, avoid soaking the wound in water (no baths, swimming, or hot tubs).

2. Dressings and Bandages

- If you have a dressing on your wound, follow your surgeon's instructions on when and how to change it.
- Keep the dressing dry and replace it if it becomes damp or soiled.

3. Monitor for Signs of Infection

- Check your wound daily for redness, swelling, increased pain, warmth, or discharge, which could indicate infection.
- Notify your healthcare provider if you notice any signs of infection, or if you have a fever.

4. Supportive Clothing

- Wear a comfortable, supportive, non-underwire bra, as recommended by your surgeon, to help reduce tension on the incision.
- Some patients find soft, loose-fitting clothing most comfortable during the healing process.

5. Avoid Heavy Lifting and Strenuous Activities

- Give yourself time to heal by avoiding any heavy lifting or strenuous activities that could strain your chest muscles.
- Your surgeon will let you know when you can safely resume certain activities, including exercise.

6. Pain Management

- Take pain medication as prescribed and use any additional pain management techniques recommended, such as using ice packs (if advised).

7. Caring for Drain Sites (if applicable)

- If you have surgical drains, keep the area around them clean and dry, and empty them as directed.
- Record the drainage amount if required, as this can help

	your doctor assess your recovery.<br>8. Follow-Up Appointments<br>• Attend all scheduled follow-up visits with your surgeon to ensure your wound is healing as expected.<br>• Your healthcare team can also provide additional instructions if needed, based on your specific surgery and health needs.<br>Always consult with your healthcare provider if you have questions about wound care, as they can provide the best guidance based on your individual recovery plan.
**7. I need surgery for breast cancer. If I have drains after the operation, how should I manage them?**	Managing drains after breast cancer surgery is important to ensure proper healing, prevent infection, and maintain comfort. Here are some general guidelines to help you care for your drains:<br>1. Drainage Care:<br>• Emptying the Drains: You'll likely need to empty your drains once or twice a day. Follow your surgeon's instructions on how often to do this. Wash your hands thoroughly before and after handling the drains to minimize infection risk.<br>• Measuring Output: Each time you empty the drain, record the amount and appearance of the fluid (e.g., color and consistency) in a log provided by your healthcare team. This information helps your doctor track your recovery.<br>• Cleaning the Drain Site: Clean around the drain site daily, following your surgeon's instructions. Gently pat dry with a clean towel.<br>2. Preventing Infection:<br>• Keep the Area Clean and Dry: Avoid submerging the drain site in water. You may be advised to take sponge baths instead of showers until the drains are removed.<br>• Monitor for Signs of Infection: Watch for signs like increased redness, swelling, warmth, drainage of pus, or a fever. Notify your healthcare provider if you notice any of these symptoms.<br>3. Pain Management and Comfort:<br>• Adjust Clothing: Wear loose-fitting clothes or a post-surgery bra with pockets to accommodate the drains, as they can feel uncomfortable if they pull or press on your skin.<br>• Reduce Pulling on the Drain Tubes: Secure the drains to your clothing or a surgical belt to avoid

	accidentally pulling or tugging on them, which can be painful.<br>4. Activity Restrictions:<br>• Avoid Heavy Lifting: Until your drains are removed, avoid activities that involve lifting heavy objects or stretching. Gentle arm movements and exercises as instructed by your healthcare team can help prevent stiffness without straining the drain sites.<br>• Stay Active Within Limits: Gentle walking is usually encouraged, as it helps with circulation and recovery, but always follow your doctor's specific guidelines for activity.<br>5. When to Remove the Drains:<br>• Drains are typically removed once fluid output decreases to a certain level (often around 30 mL or less over a 24-hour period). Your surgeon will decide when it's appropriate to remove them, usually within 1-2 weeks post-surgery.<br><br>It's always a good idea to discuss any concerns with your healthcare team, who can provide additional advice tailored to your specific recovery.
**8. I need surgery for breast cancer. When will I be able to resume physical activity after the operation, and are there any restrictions?**	After breast cancer surgery, your return to physical activity depends on several factors, including the type of surgery you undergo, any additional treatments, and your individual recovery process. Generally, here's what you can expect:<br><br>Initial Rest and Recovery<br><br>• First 1-2 weeks: You'll need to prioritize rest, allowing your body to heal from the surgery. Light activities, such as gentle walking around your home, are usually encouraged to support circulation and prevent stiffness.<br><br>Gradual Return to Activity<br><br>• 3-6 weeks: After the initial healing period, you may gradually begin light stretching and range-of-motion exercises, typically guided by a physical therapist if needed. Your healthcare team will provide specific exercises to help prevent stiffness, especially in the shoulder and arm on the surgery side.<br>• Strength exercises and lifting restrictions: Avoid lifting heavy objects and limit strength exercises involving your upper body until cleared by your surgeon. For instance, many surgeons recommend avoiding lifting anything heavier than 5-10 pounds for the first few weeks.

	Full Resumption of Exercise<br><br>• 6-8 weeks and beyond: By this stage, most people can return to moderate physical activity, such as brisk walking, cycling, and gentle yoga, depending on comfort and recovery progress. Strenuous activities like running, intense cardio, or heavy lifting may still be restricted until about 8-12 weeks or until your surgeon advises.<br><br>Key Restrictions and Considerations<br><br>• Avoid repetitive or strenuous use of the affected arm until you've fully regained strength and mobility.<br>• Look out for lymphedema signs if lymph nodes were removed, such as swelling in the arm, hand, or chest on the affected side. Gentle, supervised exercises can reduce this risk.<br>• Listen to your body and gradually build up your activity level, stopping if you feel discomfort, fatigue, or pain.<br><br>Your surgeon and physical therapist will provide a tailored plan based on your recovery needs, so follow their guidance closely.
**9. I need surgery for breast cancer. How can I prevent post-operative lymphoedema?**	I'm here to help with some guidance on preventing post-operative lymphedema. Post-operative lymphedema can occur if lymph nodes are removed or impacted during surgery, which can alter the lymphatic flow in the affected arm. Here are some strategies to minimize the risk:<br><br>1. Avoid Injury to the Affected Arm<br><br>• Protect from Cuts and Burns: Even minor cuts or burns can increase the risk of lymphedema. Use gloves when gardening, cleaning, or doing dishes, and avoid any sharp tools.<br>• Avoid Blood Pressure Readings or Needle Sticks: To prevent unnecessary pressure, avoid blood pressure measurements, IVs, or blood draws from the affected arm. Inform healthcare professionals of this precaution.<br><br>2. Practice Good Skin Care<br><br>• Moisturize Regularly: Keep your skin moisturized to avoid cracking and dryness. This helps create a barrier against infections, which can lead to lymphedema.<br>• Treat Infections Promptly: Any sign of infection, like redness, warmth, or swelling, should be addressed immediately with medical help to prevent fluid buildup.

	3. Maintain a Healthy Weight<br><br>• Balanced Diet and Regular Exercise: Weight management can lower the risk of lymphedema. Include gentle exercises (like walking or light stretching) in your routine and discuss with your healthcare provider before starting any activity.<br><br>4. Incorporate Gentle Arm Exercises<br><br>• Range of Motion and Strengthening Exercises: Gentle exercises to keep your arm flexible and to encourage lymphatic drainage can be helpful. Consult a physical therapist specializing in lymphedema for guidance on safe exercises.<br>• Avoid Overexertion: Refrain from heavy lifting or strenuous activities, especially in the weeks following surgery. Increase activity levels gradually.<br><br>5. Wear Compression Garments if Recommended<br><br>• Compression Sleeves: Some patients benefit from wearing a compression sleeve to aid lymphatic flow, especially during activities or when traveling by air. Check with a lymphedema specialist if this may be appropriate for you.<br><br>6. Stay Aware of Symptoms<br><br>• Monitor for Swelling, Tightness, or Heaviness: Any sensation of fullness, tightness, or swelling in your arm, chest, or hand could be an early sign of lymphedema. Early intervention can help prevent progression.<br><br>Lymphedema specialists, such as physical or occupational therapists with expertise in lymphatic health, can offer personalized exercises and compression therapies. Your healthcare team can also provide additional information and support to help you manage and prevent lymphedema effectively.
**10. I need surgery for breast cancer. Will I be able to eat and drink after the operation?**	After breast cancer surgery, your eating and drinking plan will be carefully managed to support your recovery. Immediately after the surgery, you may need to start with small sips of water and gradually reintroduce other liquids and foods based on how you feel and your doctor's advice. Here's what you can generally expect:<br><br>1. Right After Surgery: You might start with clear fluids like water, broth, or apple juice. If you tolerate these well, you can gradually increase to other liquids and soft foods.

	2. Gradual Return to Regular Diet: Over the next day or so, you can usually transition back to your normal diet unless otherwise directed by your healthcare team. Eating light, easy-to-digest foods can help reduce nausea and support recovery.<br>3. Stay Hydrated: Drinking plenty of fluids is important to help flush out anesthesia and medications from your system, support wound healing, and prevent constipation.<br><br>Your healthcare team will provide specific instructions tailored to your needs, so follow their advice and let them know if you experience any discomfort or nausea.
**11. I need surgery for breast cancer. How could the surgery impact my psychological well-being?**	Facing breast cancer surgery can indeed have a profound impact on psychological well-being. Here are some ways surgery may affect mental health, as well as strategies to help manage these challenges:<br><br>1. Body Image and Self-Esteem<br><br>• For many, breast surgery (particularly mastectomy or lumpectomy) can lead to changes in body image, which may affect self-esteem and confidence. The loss or alteration of a breast can create feelings of grief, shame, or a sense of loss of femininity.<br>• Coping Strategy: Seeking support groups or talking with a counselor experienced in body image concerns can help you process these feelings. Some women find comfort in breast reconstruction, while others may embrace their post-surgery body as a sign of strength and resilience.<br><br>2. Anxiety and Fear of Recurrence<br><br>• The diagnosis itself can heighten anxiety levels, and surgery might intensify worries about cancer recurrence. This fear is natural and common among cancer patients.<br>• Coping Strategy: Mindfulness practices, cognitive-behavioral therapy (CBT), and support groups can help manage anxiety. Talking to your medical team about surveillance and follow-up care can also bring a sense of reassurance.<br><br>3. Depression and Grief<br><br>• Surgery and treatment can bring on feelings of sadness, grief, or depression as you adjust to changes in your body and lifestyle. The impact of dealing with a life-threatening diagnosis may lead to emotional exhaustion or even clinical depression.

	• Coping Strategy: Staying connected with loved ones and mental health professionals can provide emotional support. Many find benefit from joining cancer support groups where they can share experiences with others facing similar challenges.<br><br>4. Impact on Intimacy and Relationships<br><br>• Changes to body image and self-esteem may affect intimate relationships. Some women worry about their partner's perception, or they may experience a decreased desire for intimacy.<br>• Coping Strategy: Open communication with your partner about your feelings and any changes can foster understanding. Counseling with a therapist who specializes in intimacy and relationship issues post-cancer can also be helpful.<br><br>5. Sense of Control and Resilience<br><br>• Surgery may bring up a feeling of lost control over your own body, which can be distressing. However, for some, undergoing surgery also leads to a newfound resilience and sense of control over their health.<br>• Coping Strategy: Focusing on self-care, healthy lifestyle choices, and activities that bring you joy can strengthen this sense of control. Working with a counselor or a support group can also be an excellent way to develop coping mechanisms and bolster resilience.<br><br>Resources<br><br>• Support Groups: Many hospitals and cancer centers offer groups specifically for breast cancer patients.<br>• Therapy: Look for counselors who specialize in cancer recovery or body image.<br>• Education: Understanding your diagnosis and treatment options can empower you, making the experience feel more manageable.<br><br>Navigating these emotions is a journey, and it's important to give yourself grace and seek support when needed. The right resources and strategies can help in maintaining and even enhancing your mental well-being through this experience.
**12. I need surgery for breast cancer. How might the surgery affect my intimacy and sex life?**	Facing breast cancer surgery can bring up a lot of concerns, including how it may affect intimacy and your sex life. Every person's experience is unique, but here are some aspects to consider and discuss with your healthcare team.<br><br>1. Physical Changes

	Breast surgery can lead to physical changes that impact how you feel about your body. For some, the loss of a breast or changes to breast shape and sensation can affect self-image and, by extension, intimacy. Nerve damage around the breast area can also lead to reduced sensation or discomfort, which may affect sexual arousal. Talking openly with your partner about any physical changes and exploring new ways of touch or connection can help you both adjust.<br><br>2. Emotional and Psychological Impact<br><br>The emotional impact of a cancer diagnosis and the stress of treatment can sometimes decrease sexual desire. Anxiety, depression, or concerns about how your body has changed can make it challenging to feel intimate. Support groups or counseling can provide a safe space to talk through these emotions, and many people find that open communication with their partner helps as well.<br><br>3. Hormonal Changes<br><br>If your treatment includes hormone therapy, it can cause symptoms like vaginal dryness, hot flashes, and fatigue, which may impact your sex life. A lubricant or vaginal moisturizer can help manage dryness, and lifestyle adjustments can often help with other symptoms. Speak with your doctor about managing these side effects to make intimacy more comfortable.<br><br>4. Practical Tips for Maintaining Intimacy<br>• Take it slowly: Give yourself time to heal and adjust after surgery before diving back into sexual activity.<br>• Communicate openly: Share your feelings and concerns with your partner to maintain emotional closeness.<br>• Explore new ways to be close: Kissing, cuddling, or other non-sexual touch can help maintain intimacy and may gradually ease you back into feeling comfortable with your body.<br><br>Remember, there are many ways to experience closeness and affection. Your feelings and needs may change over time, and that's okay.
**13. I need surgery for breast cancer. How can I manage the pain during the postoperative period?**	Managing postoperative pain after breast cancer surgery is crucial for recovery and overall well-being. Here are some strategies to help you manage pain during this time:<br><br>1. Medications: Your surgical team will likely prescribe pain relievers, which may include NSAIDs (like ibuprofen), acetaminophen, or stronger medications if needed.

	Follow the prescribed dosage and discuss any side effects with your healthcare provider.<br>2. Cold Therapy: Applying a cold pack to the surgical area for short periods (usually 10-15 minutes) can help reduce swelling and numb pain. Make sure to place a cloth between the cold pack and your skin to prevent frostbite.<br>3. Gentle Movement and Stretching: Your healthcare team might recommend specific arm and shoulder exercises to prevent stiffness and maintain range of motion. Start these only when advised, as they can also help relieve discomfort over time.<br>4. Breathing and Relaxation Techniques: Deep breathing exercises and mindfulness techniques can help calm your body and reduce the perception of pain. Guided imagery or meditation apps can be helpful tools.<br>5. Positioning: When lying down, use pillows to support your arm and keep your chest elevated. This can alleviate some pain and pressure in the chest area.<br>6. Complementary Techniques: Techniques like acupuncture, massage, and transcutaneous electrical nerve stimulation (TENS) may be options for some patients. Consult your healthcare provider to see if these are appropriate for you.<br>7. Stay Hydrated and Nourished: Proper nutrition and hydration support healing and can also help with medication effectiveness and side effect management.<br>8. Pain Management Plan: Have an open discussion with your healthcare team about a pain management plan tailored to your needs. This should include what to expect as you recover, pain-relief options, and any modifications needed over time.<br><br>If your pain intensifies or you notice any unusual symptoms, contact your medical team, as this may require a change in your pain management approach or a check-up to ensure there are no complications.
**14. I need surgery for breast cancer. How long will I need to stay in the hospital after the operation?**	The length of your hospital stay after breast cancer surgery depends on the type of surgery you undergo and your specific health needs. For common types of breast surgery:<br><br>1. Lumpectomy (Breast-Conserving Surgery): This often allows you to go home the same day, as it's typically an outpatient procedure.<br>2. Mastectomy (Removing the Entire Breast): A hospital stay of 1 to 2 days is common, though some mastectomies can be done as outpatient procedures, especially if no reconstruction is involved.<br>3. Mastectomy with Reconstruction: If you're also having

	reconstructive surgery, you may need to stay in the hospital for 2 to 3 days, depending on the type of reconstruction (such as implants or tissue flap procedures).<br><br>Your surgical team will monitor your recovery, pain management, and any drains placed during surgery, which could influence your discharge timing. Before you leave, your healthcare provider will go over wound care, activity limitations, and follow-up care to ensure a safe and comfortable recovery at home.
**15. I need surgery for breast cancer. Will I need follow-up appointments after the procedure?**	Yes, follow-up appointments are essential after breast cancer surgery. These visits help monitor your recovery, manage any side effects, and ensure the cancer hasn't returned. Here's what you can expect from these follow-up appointments:<br><br>1. Initial Follow-Up: Typically, a few weeks after surgery, you'll have an appointment to check the healing of your incision, discuss any pain or discomfort, and review your pathology results if a biopsy was done.<br>2. Ongoing Check-Ins: In the months and years following surgery, regular check-ups will allow your healthcare team to monitor for any signs of recurrence and help manage any long-term effects of the treatment, such as lymphedema or changes in your physical and emotional well-being.<br>3. Screening and Imaging: Depending on your specific case, you may need periodic imaging tests, like mammograms or ultrasounds, to check for changes in the breast tissue.<br>4. Supportive Care: Follow-up appointments may also include referrals for physical therapy, counseling, or support groups, which can be beneficial for emotional and physical recovery.<br><br>Your healthcare provider will personalize a follow-up schedule based on your treatment and recovery needs. Keeping up with these visits can be a critical part of your recovery journey.

Table S2. Accuracy (4-point Likert scale) and readability (FKGL) scores for each individual response generated by ChatGPT-4o.

Response	Accuracy	Readability	Reading Difficulty	Grade Level
**N.1**	1	11.3	Average	High School
**N.2**	1	9.5	Average	High School
**N.3**	1	10.9	Average	High School
**N.4**	1	11.7	Average	High School
**N.5**	1	9.3	Average	High School
**N.6**	1	8.7	Average	Middle School
**N.7**	2	9.2	Average	High School
**N.8**	1	12.4	Advanced	Middle School
**N.9**	1	11.3	Average	High School
**N.10**	1	10.9	Average	High School
**N.11**	2	11.2	Average	High School
**N.12**	1	10.5	Average	High School
**N.13**	1	11.9	Average	High School
**N.14**	2	13.6	Advanced	Middle School
**N.15**	2	13.3	Advanced	Middle School
